# An investigation into the diagnostic accuracy, reliability, acceptability and safety of a novel device for Continuous Ambulatory Vestibular Assessment (CAVA)

**DOI:** 10.1038/s41598-019-46970-7

**Published:** 2019-07-18

**Authors:** John S. Phillips, Jacob L. Newman, Stephen J. Cox

**Affiliations:** 1 Norfolk and Norwich University Hospital, Department of Otolaryngology, Norwich, NR4 7UY United Kingdom; 2 University of East Anglia, School of Computing Sciences, Norwich, NR4 7TJ United Kingdom

**Keywords:** Computer science, Diagnosis, Eye manifestations, Medical research

## Abstract

Dizziness is a common condition that is responsible for a significant degree of material morbidity and burden on health services. It is usually episodic and short-lived, so when a patient presents to their clinician, examination is normal. The CAVA (Continuous Ambulatory Vestibular Assessment) device has been developed to provide continuous monitoring of eye-movements, allowing insight into the physiological parameters present during a dizziness attack. This article describes the first clinical investigation into the medical and technical aspects of this new diagnostic system. Seventeen healthy subjects wore the device near continuously for up to thirty days, artificially inducing nystagmus on eight occasions. 405 days’ worth of data was captured, comprising around four billion data points. A computer algorithm developed to detect nystagmus demonstrated a sensitivity of 99.1% (95% CI: 95.13% to 99.98%) and a specificity of 98.6% (95% CI: 96.54% to 99.63%). Eighty-two percent of participants wore the device for a minimum of eighty percent of each day. Adverse events were self-limiting and mostly the consequence of skin stripping from the daily replacement of the electrodes. The device was shown to operate effectively as an ambulatory monitor, allowing the reliable detection of artificially induced nystagmus.

## Introduction

**Figure 1:**
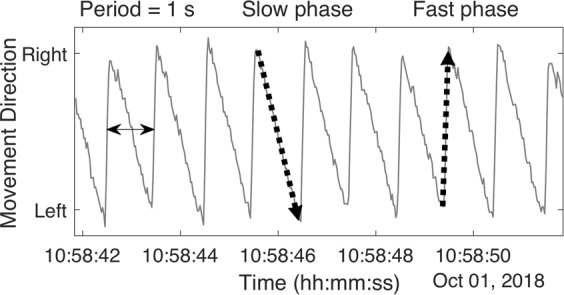
A diagram showing right-beating nystagmus induced by watching a left-moving dot on a VR headset. The fundamental frequency of the signal shown is 1 Hz. A double-headed arrow marks the waveform period, which corresponds to the time taken for the dot to travel once across the headset screen. Examples of the slow and fast phases of the nystagmus are indicated by downward and upward pointing arrows.

Dizziness is a common, non-specific complaint. In England and Wales, eight out of every 1,000 patients are likely to consult with their General Practitioner complaining of dizziness every year^1^. One in four in the community have ‘significant’ dizziness at any given time^2^. Between 13% and 16% of dizzy patients are referred for a specialist opinion; up to 36% of these referrals are to an ENT clinic^1,3^. Dizziness is the most common reason for a physician to visit a patient over the age of 75 years old^4^, and one-third of adults over the age of 65 years experiences at least one fall each year^5^. A common cause of dizziness is a malfunction in the vestibular system which is located in the inner ear^6^. There is an intimate relationship between eye movements and the inner ear which is mediated by a neural reflex called the vestibulo-ocular reflex (VOR), the main purpose of which is to stabilise images on the retina during head movements. Because of this relationship, episodes of dizziness caused by inner ear malfunctions are often accompanied by an abnormal jerking eye movement called *nystagmus* (Fig. 1). Therefore, eye movements are key in assessing the function of the vestibular system^7^.

The onset of dizziness accompanying vestibular dysfunction is often unpredictable and episodic, making it unlikely that an episode of dizziness will coincide with a patient’s visit to a specialist^8^. Although many traditional and contemporary balance tests are available, they only provide a snapshot of vestibular function in the absence of an actual ‘dizzy attack’^9^. Two conditions which attract considerable attention in the clinical and research communities are Ménière’s disease and Vestibular Migraine. The causes of these conditions are not fully understood and their assessment is challenging^10^. Both of these conditions may result in dizziness lasting for many hours, but patients may be asymptomatic for days, weeks or even months between attacks. Conditions other than Ménière’s disease and Vestibular Migraine can present in an equally sporadic manner.

**Figure 2:**
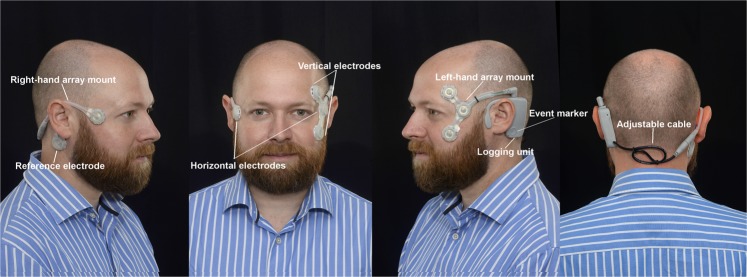
The CAVA device as it appears when worn on the face. The device comprises a logging unit which sits behind the left ear, and five electrode pads which adhere to the face.

The Continuous Ambulatory Vestibular Assessment (CAVA) device is a novel, medical device for recording eye movements continuously over a period of thirty days (Fig. 2). The device is composed of two main components: a bespoke single-use sensor array that adheres to the participant’s face, and a small, reusable, electronic logging unit. The device is lightweight and durable, and can be worn continuously throughout the day and night. While worn, the device continuously records horizontal and vertical eye movements and an accelerometer captures head movement. Eye movements are captured passively by way of the dipole potential (the corneo-retinal potential) that exists between the front and the back of the eyeball, which can be used as a proxy for eye movement. CAVA has been developed as part of a program of work funded by the UK Medical Research Council to develop a diagnostic system to aid the diagnosis of dizziness. The system itself comprises the CAVA device (designed and produced by Wright Design Limited, in Cambridge, UK) and separate computer algorithms for detecting short periods of nystagmus contained with days’ worth of normal eye-movement data (developed at the University of East Anglia, Norwich, UK).

CAVA is intended to be worn in non-clinical settings by patients suffering from suspected dizzy attacks. At the end of a thirty day monitoring period, the data captured by the device would be downloaded and analysed offline by computer algorithms for signs of nystagmus. Episodes of dizziness detected by the system would be presented to a clinician for further analysis and could be used to support a diagnosis. Nystagmus may be classified in a number of ways, including by duration, frequency, beat direction and other characteristics^11^. Nystagmus is divided into a *slow* and *fast* phase, corresponding to the eyes drifting slowly in one direction, before quickly moving in the opposite direction. The nystagmus shown in Fig. 1 is *right*-*beating*, as defined by the direction of the fast phase. Using the data captured by the CAVA device, we expect to be able to determine the aetiology of dizziness episodes. Whilst peripheral vestibular nystagmus is typically horizontal in nature (or torsional in Benign Paroxysmal Positional Vertigo); downbeat and upbeat nystagmus can be identified in a large number of conditions from outside the vestibular system^12^. For example, hyperventilation or intracranial hypotension are associated with characteristic forms of nystagmus, such as downbeat nystagmus^13–15^. Parallels can be drawn between the application of the CAVA device and the 24-hour ECG tape that is used to identify cardiac arrhythmias^16,17^ and ambulatory EEG^18–20^.

As the CAVA device is an entirely novel device for long-term, ambulatory monitoring, there is little previous literature with which comparisons can be made. However, a portable recorder for examining nystagmus in non-clinical settings was described several decades ago^21^. The prototype described could record two-channels of eye movement for a period of ten minutes, although this was intended to be increased to eighty-minutes in the following version. Owing to the limited data storage, the device was intended to be worn and activated by a subject at the onset of an episode of dizziness. Therefore, the device relied on the wearer to correctly identify the onset of an attack, and on them being capable of applying the device whilst experiencing symptoms of dizziness. The CAVA device represents a significant improvement on that device in terms of the duration of data capture and portability. If the CAVA system can detect nystagmus from its ambulatory data, this could pave the way to overcome many of the shortfalls of existing approaches to vestibular assessment, potentially reducing the number of hospital visits and unnecessary tests, and providing faster diagnoses and access to treatment.

There is much literature addressing the task of automatically identifying nystagmus from short-term eye-movement data^22–24^. However, there is no previous work focused on the analysis of long-term eye-movement data, as CAVA is the first device to provide such data. Previous automated approaches have exploited features of eye movement which, although well suited to short-term nystagmus classification in laboratory conditions, are unlikely to generalise well when applied to days’ worth of highly diverse eye movement data. For example, nystagmus detection usually involves identifying saccades (the fast phase of nystagmus) using eye movement velocity. A day’s worth of eye-movement data contains vast quantities of normal saccadic eye movement, making reliable discrimination more difficult. As the primary objective of this study is to demonstrate that nystagmus can be detected from the long-term data captured by the CAVA device, this study poses a significant and novel challenge to the field of computer science.

This article describes the first formal clinical investigation undertaken to evaluate the CAVA device in healthy volunteers who wore the device for up to thirty days. The primary objective of this investigation was to demonstrate the diagnostic accuracy of the system to detect artificially induced nystagmus. To meet this objective, bespoke computer algorithms were developed to analyse the data offline, in a blinded recognition experiment undertaken at the end of the trial. The algorithms were required to demonstrate a diagnostic sensitivity and specificity of 95% or greater for the task of identifying the dates on which participants induced nystagmus. To recreate the nystagmus eye movements produced by dizzy patients, each healthy participant watched a thirty-second nystagmus-inducing video on up to eight pre-determined dates. The other aims of the investigation were to evaluate device reliability, safety, acceptability and user experience. To meet objectives in these areas, the device had to provide less than 5% non-useful data for each participant, for each day of the trial, and across the entirety of the trial. The event-marking, timestamping and accelerometer functions of the device had to function correctly. Per participant, less than five data drop-outs per day were permitted, and less than sixty across the whole trial. In terms of compliance, 80% of participants were required to comply for a minimum of 80% of the complete trial, and for a minimum of 80% of each day. There were no objectives associated with user experience and safety, but the data is presented for clarity and a qualitative analysis is provided. Following the completion of this trial, we intend to undertake a second investigation involving patients suffering from vertigo.

## Methods

### Study design

**Figure 3:**
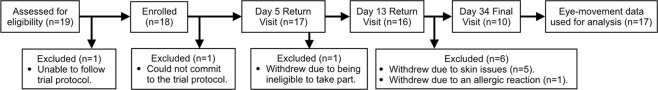
Consort diagram showing the flow of study participants through the trial.

This study was a single-arm, blinded, clinical investigation undertaken at the Norfolk and Norwich University Hospital, in the United Kingdom. Nineteen healthy participants were screened and the CONSORT diagram in Fig. 3 shows the flow of participants through the trial. The primary aim of this study was to show that short periods of artificially induced nystagmus could be detected from within days’ worth of normal eye movement data captured by the CAVA device. The study was registered on clinicaltrials.gov (NCT03661762) on 7th September 2018. The Medicines and Healthcare products Regulatory Agency (MHRA) was notified of the investigation, who reviewed and approved the trial. All trial processes and trial documentation, including the protocol, were reviewed and approved by the London-Dulwich Research Ethics Committee (IRAS: 240847). The study was carried out in accordance with the relevant guidelines and regulations. A recruitment target of fifteen participants and a minimum number of positive samples (dates containing nystagmus) were determined by a medical statistician (see *Statistical Analysis* section). Prior to the start of the trial, the first participant was designated as a *dry*-*run*. The *dry*-*run* participant would be excluded from the final, blinded analysis, and would provide the opportunity for refinements to be made to the device and trial processes in the event that any minor operational issues were encountered.

The study was designed around a blinded nystagmus-recognition task, in which bespoke computer algorithms for detecting nystagmus were applied to the data captured during the trial. As this was a healthy volunteer trial, participants did not experience episodes of dizziness. To simulate dizziness, each participant induced physiological, horizontal nystagmus by watching a video on a portable screen. Participants were expected to wear the device for thirty days, and were instructed to induce nystagmus on eight occasions. At the end of the trial, the data captured was downloaded, randomised (See section *Data Randomisation*, *Blinding and Analysis*) and then computer algorithms were applied to the data to detect the dates on which nystagmus was induced (See section *Nystagmus Detection Algorithm*). In addition to this, data relating to device reliability, participant compliance and user experience were captured during the trial.

Healthy individuals were recruited through a poster campaign at the University of East Anglia, in the United Kingdom. Participants were recruited according to availability and no sampling of the population took place. The population of potential participants consisted primarily of university-age students and also of working-age university staff. Potential participants approached the study team and were provided with an information sheet detailing the requirements, benefits and risks of participation in the study. Interested participants attended a consenting visit at the hospital, where informed consent was obtained for all participants. Once consented, eligibility criteria were checked (see *Inclusion and Exclusion Criteria* section), and successfully screened participants were enrolled onto the trial. Participants were trained regarding all trial procedures, and were provided with one device and enough single-use electrode mounts to replace them daily, for thirty days. Participants were allotted one hour each day to shower and to renew the electrode mounts. The electrodes contained within the mounts were the Covidien Kendall H124SG electrodes, which are small ECG/EMG electrodes. Each device was activated prior to the participant leaving the clinic, and it recorded data continuously until deactivated by a member of the study team at the end of the trial.

Participants returned to the hospital on day five to make sure that they were not experiencing any difficulties, and that they had not experienced an allergic reaction to the materials in the electrode pads. On day thirteen, participants returned so that the device’s battery could be renewed and to perform an interim data download. The device’s event marker was also pressed during this visit, providing data to allow the device’s timestamping and event marking functions to be assessed at the end of the trial. The final return visit was on day thirty-four, when they returned the device and were debriefed from the trial.

During the final visit, participants completed a short questionnaire, designed to gather feedback on the device and the trial. The questionnaire comprised ten questions, with eight requiring scale-based responses, and two requesting a written comment. Space was provided for participants justify all ratings given. Participants also performed a series of head movements, designed to assess the functionality of each device’s accelerometer. Three separate head movements were performed, at both a slow and a fast speed.

On eight occasions during the trial, each participant induced physiological, horizontal nystagmus by watching a video on a portable screen. Each participant was randomly allocated one of six videos. The selection of videos included three different speeds of moving dot, and the dots either moved in a leftward or rightward direction. Participants watched the video whilst remaining stationary for the first four days, and whilst walking gently on the spot for the remaining four days. Prior to watching the video, participants were instructed to press the event marker button on the CAVA device, which added a timestamped annotation to the device data. This annotation was used by a member of the study team to verify that the video had been viewed (See section *Data Randomisation*, *Blinding and Analysis*). After the trial, the horizontal eye movement data was extracted, randomised and then a blinded investigator used bespoke computer algorithms to predict the presence or absence of nystagmus in each day’s worth of data. The eye movement data from the first participant (*dry*-*run*) were not included in the final, blinded recognition task. The sensitivity and specificity of the algorithm’s predictions were determined by a medical statistician using the number of true positive, false positive, true negative and false negative detections.

### Inclusion and exclusion criteria

Inclusion criteria were healthy volunteers who were (a) aged 18 and over (b) were able to commit to thirty days of continuous wear of the trial device (c) owned a telephone. Exclusion criteria were (a) potential participants who had a history of dermatological disease or damage around the forehead (b) potential participants who had an allergy to plasters and/or medical adhesives (similarly to materials used in the device) (c) a history of dizziness, vertigo, balance disorders, or syncope (d) history of hypertension or cardiac problems (uncontrolled, acute or de-compensated phase) (e) history of ear disease, or previous ear surgery (f) history of psychotic/neurotic disorders or epilepsy (g) history of eye disease, or previous eye surgery (h) pregnant or nursing mothers (i) unable to follow the testing protocol.

### Data randomisation, blinding and analysis

At the end of each participant’s trial, the data from their device was downloaded by the research team. None of the device data contained participant identifiable information. The data was first converted from the compact representation used during storage to a human readable format (A Microsoft Excel compatible file). Each participant’s data was re-segmented into thirty full calendar days’ worth of data and the data from a single day constituted a file. Only the data corresponding to the horizontal eye movement was retained, and all other data was discarded, including the event marker data. The first and last days of the trial (days one and thirty-one) were half days, and the data from these days were combined to approximate one full day. Multiple data files within a single day were also combined (e.g. on day thirteen, resulting from the battery change). Finally, re-segmentation also adjusted for Daylight Saving. Once resegmented, the data was uploaded to a secure server at the University of East Anglia.

The unblinded investigator was responsible for randomising the trial data and for maintaining the blinding from the blinded investigator. Before randomisation, the unblinded investigator manually reviewed the trial data for evidence that the device had recorded an informative signal (i.e. not simply a flat line), and also examined the signals for evidence of the characteristic nystagmus waveform on the dates and times logged in the participant’s trial diaries and using the event marker data. Based on these observations, each file was assigned a label indicating the presence of absence of nystagmus in the file (the ground-truth label).

After labelling, the unblinded investigator assigned each of the individual days a randomised filename. Using a coding sheet, only the unblinded investigator was able to determine the origin (participant ID and date) for each of the randomised files. The data was then analysed offline by the blinded investigator, using a specially-designed computer algorithm (see *Nystagmus Detection Algorithm* section). This algorithm produced a list of files predicted to contain a horizontal nystagmus signal, and corresponding values of the signal’s beat direction and beat frequency. The unblinded investigator compared the predicted dates from the algorithm with the ground-truth dates in the coding sheet. Finally, the results were analysed by the project’s designated medical statistician to determine the diagnostic accuracy of the results obtained. This analysis consisted of calculations of sensitivity and specificity, as described in the *Statistical Analysis* section. The blinding was formally lifted once the final analysis was complete.

### Nystagmus detection algorithm

A nystagmus detection algorithm was developed to detect automatically the periods of horizontal nystagmus contained within the trial data. The system used to analyse the trial data employed an ensemble of machine learning techniques, built using frequency domain recognition features, and consisting of a multi-stage recognition process in which potential nystagmus candidates were accepted or rejected based on several heuristic metrics.

For feature extraction, a Fast Fourier Transform (FFT) was used to transform the time series data into the frequency domain. The data was processed using a sliding window with an 80% overlap and a window length of 500 samples, producing frames representing the positive frequency amplitudes. 82 points of the FFT were retained, corresponding to frequencies between 0.25 Hz and 7 Hz, spaced at 0.083 Hz intervals. These parameters, and others used in the system, were selected based on experiments on preliminary data recorded prior to the trial.

Three separate machine learning classifiers were trained using the features derived from the training data: A Support Vector Machine (SVM)^25^, a Linear Discriminant Analysis transformation (LDA)^26^ and an ensemble classifier of boosted trees^27^. These classifiers were trained to identify nystagmus, irrespective of direction and speed. During testing, the outputs of these classifiers were combined by way of a majority voting system, and the output was smoothed using a *sieve* filter^28^. Combining the classifier outputs in this way, often referred to as an ensemble of classifiers, has been shown to be an effective approach to time series classification^29^.

Lastly, a validation step occurred on each run of consecutive frames classified as nystagmus. In this step, Dynamic Programming (DP) was used to compare the time series signal to a sawtooth waveform. The output of this process was a value that quantified the degree to which the eye movement signal matched a sawtooth waveform. Any data file containing positively classified frames was considered as a positive detection of nystagmus. Each detected nystagmus event was further classified according to its beat direction and beat frequency.

To classify the nystagmus beat direction, the velocity signal was calculated from the time series waveform, as the velocity of the signal provides useful information which can be used to determine the beat-direction of the nystagmus. For example, the slow phase of a normal sawtooth wave contains mostly negative velocities, while for a mirrored sawtooth, the signs are inverted. Each frame was assigned a weighting (between −1 and 1) reflecting the proportion of negative and positive velocities. The average weighting within the event was used to classify the direction of the nystagmus. For the classification of nystagmus frequency, the FFT was calculated for each event. From the FFT output, the modal frequency bin was identified, and the frequency of the nystagmus was classified as the closest class to that bin. In the case of a tie between bins, the highest neighbouring bin was found and the decision was based upon that frequency instead.

### Statistical analysis

The success of a diagnostic device to diagnose disease can be appraised by calculating values for sensitivity and specificity. This trial has been designed to exceed the minimum number of nystagmus days and minimum number of total days required to demonstrate with statistical significance a sensitivity and specificity of over 95%. This standard was determined from data in the field of ambulatory cardiac monitoring^30^. The calculations below were performed by a medical statistician to determine the minimum number of independent tests for the nystagmus detection algorithms to demonstrate the required level of diagnostic sensitivity and specificity.

In the usual way, we define:

- *Sensitivity* = (# *days on which nystagmus was detected and present*)/(# *days on which nystagmus was detected*)
- *Specificity* = (# *days on which nystagmus was not detected and not present*)/(# *days on which nystagmus was not detected*)

Each file was a day’s worth of data and contained either zero or one nystagmus event. We assumed independence of these events as the data was randomised prior to analysis. The primary objective for our algorithm was to demonstrate a sensitivity >95% and a specificity >95% at a confidence level of 95%. For a mean sensitivity and specificity of 98% with a maximum marginal error of 3%, for constructing the 95% confidence interval (0.9442 to 1.000), a sample size of 85 positive and 85 negative events is required (Table 1). In practice, we recorded 112 positive events and 293 negative events.

**Table 1:** Minimum number of positive and negative samples required to demonstrate a sensitivity and specificity of over 95%.

		Device Prediction		Total
		TRUE	FALSE	
Ground-truth	TRUE	83 (97.7%)	2 (2.3%)	85
	FALSE	2 (2.3%)	83 (97.7%)	85
	Total	85	85	170

## Results

Nineteen participants were screened and eighteen participants were enrolled onto the trial. One participant failed screening as they were unable to follow the training protocol. One participant was withdrawn having worn the device for less than twelve hours, as it became apparent that they were unable to adhere to the trial protocol. For this reason, we have excluded this participant from all further analyses, giving a total of seventeen participants analysed. Of these seventeen participants, seven withdrew before completion of the full trial (see *Compliance* section). The first participant was recruited as a *dry*-*run* participant, allowing the device to be evaluated in full prior to deployment of the remaining participants. There were no deviations from the trial protocol which could have affected the scientific value of the study. A summary of the demographics of the study participants is presented in Table 2. A broad range of ages were recruited, which is likely to be consistent with the distribution of ages present in a university population of staff and students. Nearly twice as many females than males were recruited. This was not by design, and appeared to be due to the increased willingness of females to volunteer for the study. The following sections report the results of the trial in the areas of diagnostic accuracy, device reliability, safety, acceptability and participant feedback.

**Table 2:** Demographics of the participants enrolled in the CAVA: Healthy Volunteer Trial.

Characteristic	Value (n = 17)		
Age (yrs.)	**18 to 25**: 6	**26 to 35**: 5	**36 to 45**: 3
	**46 to 55**: 2	**56 to 65**: 1	
Gender (frequency)	**Male**: 6	**Female**: 11	

### Diagnostic accuracy

Each day of data captured from a participant was either a “non-nystagmus” day or a “nystagmus” day, determined by whether or not the subject had induced nystagmus on that day. A computer algorithm used to identify the nystagmus days achieved a detection sensitivity of 99.1% (95% CI: 95.13% to 99.98%) and a specificity of 98.6% (95% CI: 96.54% to 99.63%), when applied to the randomised trial data (see *Statistical Analysis* section for definitions of sensitivity and specificity). Table 3 provides the classification results of this task in terms of true positive, false positive, true negative and false negative detections. The single false negative was found to contain a noise impulse in the nystagmus signal, caused by the participant pressing the device’s event marker button midway through inducing nystagmus. The false positives were found to generally be periodic in nature, and observation of the concurrent accelerometer data showed that they might be due to physical activities such as running on a treadmill.

**Table 3:** Confusion matrix for the nystagmus detection task.

		Ground Truth		Total
		Present	Absent	
Predicted	Present	111	4	115
	Absent	1	289	290
	Total	112	293	405

A more detailed recognition task was conducted on the 111 correctly identified nystagmus events (Tables 4 and 5). This analysis included the classification of nystagmus beat direction and frequency. Nystagmus beat direction is defined by the direction that the eyes move during the fast phase. For the task of classifying beat direction, an accuracy of 99.10% (one incorrect classification) was attained. For the three-class recognition task of classifying the fundamental beat frequency, the accuracy was 98.20% (two incorrect classifications). These values indicate that, using data captured by the CAVA device, a minimum duration of thirty seconds of visually-induced nystagmus can be identified automatically with a high degree of accuracy, including some more detailed characteristics of the associated waveforms.

**Table 4:** Confusion matrix for the classification of beat direction.

		Ground Truth		Total
		Left	Right	
Predicted	Left	52	1	53
	Right	0	58	58
	Total	54	61	111

**Table 5:** Confusion matrix for the classification of beat frequency.

		Ground Truth			Total
		0.8 Hz	1.0 Hz	1.2 Hz	
Predicted	0.8 Hz	35	1	0	36
	1.0 Hz	0	35	1	36
	1.2 Hz	0	0	39	39
	Total	35	36	40	111

### Hardware reliability

The majority of trial participants (12 of 17) experienced no technical difficulties or hardware issues relating to the use of the CAVA device. No issues were reported relating to battery life or device usability. The device’s event marking function was found to operate as expected, but each device displayed a timestamping clock drift of up to twenty-one minutes per day. This drift is a small issue relating to the labelling of the data, and did not cause any loss of device data.

The devices’ accelerometers functioned as intended, with nearly all head movements being easily discernible from the data captured. However, identification of head turning was slightly reduced compared to the other head movements, perhaps due to the staged nature of the task (sitting upright in a chair, in a clinical setting) and because this motion is predominantly rotational, rather than accelerative along a linear axis.

During the first participant’s trial (*dry*-*run*), an issue was identified relating to the cable for the right-hand electrode mount. Due to compression of the cable during sleep and the repeated reapplication of the device, the cable could twist over time, compromising the device’s internal solder joints. This issue caused a data drop-out lasting approximately three hours. Following discovery of this issue, a mechanical fix was implemented on all devices, and it did not re-occur.

In total, four technical issues arose during deployment of the remaining participants. One participant reported that their device was sporadically indicating a possible “lead-off event” (device not properly attached to the face). This participant was advised to apply a new set of mounts, which resolved the issue. Inspection of the mounts that were removed revealed that a single solder point had failed. This issue caused a sporadic loss of vertical eye movement data, but all other data was intact. This issue occurred only once.

Three malfunctions occurred leading to a short-term data drop-out. One participant reported that their device was permanently displaying an error status. A software error was found to have occurred, causing the device to stop logging. The device was repaired and then resumed normal functioning. Another participant reported a similar issue, but around nine hours after they had stopped wearing the device due to skin issues. Lastly, a participant reported that, having reapplied their device, it was unresponsive to button presses. This device was restarted and resumed logging. All data captured during the trial was useful, providing valuable information about eye and head movement. The maximum number of drop-outs per day was one, with three occurring in total, reported by three separate participants (following the *dry*-*run*). These values are within the maximum limits for non-useful data and drop-outs as specified in our trial objectives.

### Acceptability

**Figure 4:**
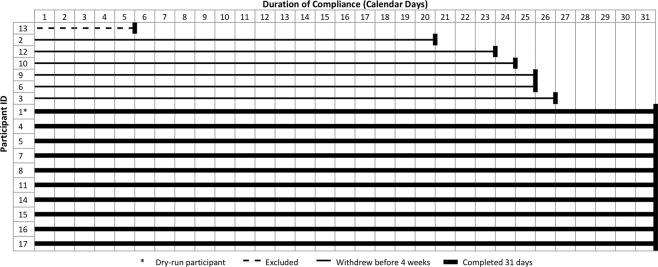
Trial compliance per participant, up to and including completion or withdrawal.

Ten participants completed the thirty day trial in full, and all ten wore the device on every full day of their trial. Figure 4 displays the compliance per participant, shown as the number of calendar days completed before withdrawal from, or completion of the trial. Each participant was expected to wear the device across thirty-one calendar days, applying it for the first time in the morning of day one and removing it for the final time on day thirty-one. The mean trial duration across participants was twenty-seven days (excluding participant thirteen and taking the full trial duration to be thirty days).

Seven participants withdrew prior to day thirty-one. Of these seven, one participant was asked to withdraw having revealed that they met one of the trial’s exclusion criteria. The remaining six participants withdrew because of the onset of skin issues resulting from the use of the device’s electrodes (see *Safety* section). Of the six participants who withdrew because of skin issues, all complied fully up to the point that they were withdrawn through mutual agreement with the study team (three participants), or withdrew prior to contacting the project team (three participants).

Daily compliance was calculated using the participants’ trial diaries (Supplementary Fig. S1). For these calculations, a day was taken as twenty-three hours, as participants were allowed to remove the device for an hour each day in order to shower. 82% of participants (14 of 17) complied for more than 80% of each day. 90% (9 of 10) who completed the trial in full, were fully compliant for each day of the trial. In cases where compliance was not full, participants had removed the device because of the onset of skin issues or had been instructed to remove it by the study team.

### Safety

Seven unique adverse events were reported during the trial, two of which were unrelated to the device, and one of which (disturbed sleep) was tolerated by the participant who reported it. The remaining four adverse events were related to the onset of a self-limiting skin issue resulting from near-continuous wear of the device’s electrode pads. Of the seventeen participants analysed, nine (53%) experienced a degree of skin reddening. Of these nine participants, six (67%) withdrew from the trial as a result of the reddening, and three were able to continue by repositioning the electrode sites to avoid the affected skin. Two of the nine participants (22%) revealed a history of dermatological disease after the reddening had started. The project’s designated dermatologist proposed that this issue was an exaggerated form of the appearance one would expect to see in an individual who repeatedly reapplied a sticking plaster. The cause was hypothesized to be a combination of the trauma of electrode removal and the long-term occlusion of the skin. One participant experienced an allergic reaction to the materials in the electrode pads.

Reporting of skin redness was always delayed, and was therefore correlated with wear duration and the frequency of pad replacement. The first participant (*dry*-*run*) reported reddened skin on day four, and following this, subsequent participants were provided with a medical adhesive remover, to ease removal of the electrodes. Of the remaining eight participants who experienced a skin issue, seven reported redness on or after day nineteen and the minimum duration before reporting increased to thirteen days. These results suggest that the adhesive remover may have delayed the onset of skin related issues. The issue was self-limiting and resolved quickly and fully once the participants had stopped reapplying the device. No serious adverse events were reported during the trial.

### Participant Feedback

**Figure 5:**
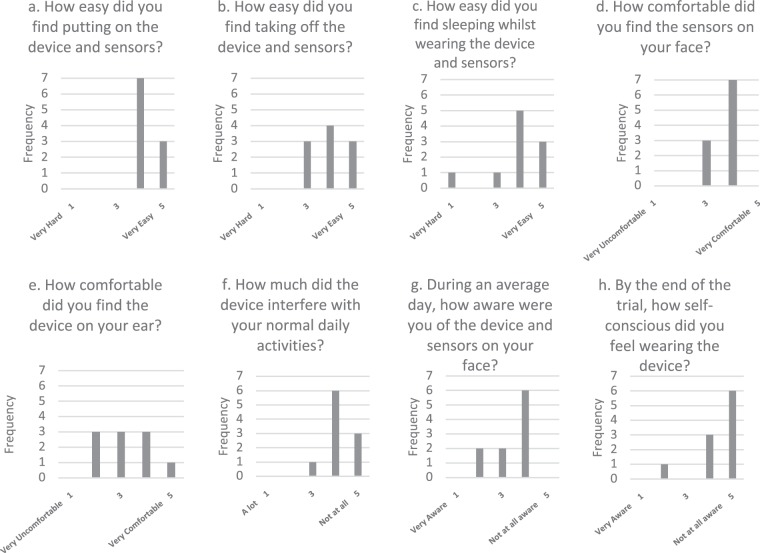
Trial questionnaire results. Only participants who completed the trial in full were asked to complete the questionnaire.

Each participant who completed the full trial completed a questionnaire designed to gather feedback on their experiences during the trial (Supplementary Fig. S2). Analysis of their responses revealed largely positive feedback (Fig. 5). In terms of device usability, all participants found the device to be both easy to apply and to remove from the face (Fig. 5a,b). The majority of participants found it easy to sleep whilst wearing the device (c). Participants generally found the device to be comfortable to wear (d), with some reporting more discomfort from the device sitting over the ear (e). With respect to the social implications of wearing the device, all participants reported that the device did not interfere greatly with their normal daily activities (f), and that they were generally unaware of the device during an average day (g). A majority said that they did not feel self-conscious whilst wearing the device (h).

## Discussion

This is the first investigation of a novel, ambulatory device for recording eye movements over a period of thirty days. As such, the data collected and results obtained are equally novel. Early work into similar technology was limited by the capability of technology at that time^21^. Specifically, previous attempts were bulky, restricted the wearer’s vision, and did not offer sufficient data storage to be used as a long-term, ambulatory device. Advancements in computer processing, data storage, device miniaturisation and battery life have allowed the development of a highly portable device which can record vast amounts of information. The usefulness of the data captured is demonstrated by the ability of our computer algorithms to attain high levels of diagnostic accuracy for the task of identifying short periods of visually-induced nystagmus from within days’ worth of normal eye movement data (99.1% sensitivity and 98.6% specificity). The algorithms were also able to distinguish between left and right-beating nystagmus, and different speeds of nystagmus, with similarly good results.

The device was shown to be very reliable, with only a handful of technical issues arising during 405 days of deployment. Two of the issues that arose were the result of simple mechanical failures, which were easily repaired. The other issues were related to device software, and were easily overcome by restarting the logging-unit. The device’s status-checking functionality proved vital to the early identification of these issues, and helped to minimise the duration of the resulting data drop-outs. The ancillary functions of the device, such as the accelerometer, event marking and timestamping, were shown to function as intended, although the accuracy of the timestamping was inadequate. The cause of this software issue has since been identified and rectified. In summary, the device has fulfilled the trial objectives which included the maximum amount of non-useful data and the number of data drop-outs permitted during the trial, as well as the correct functioning of the device’s accelerometer and event marker.

Participant compliance throughout the trial was generally high, meeting the trial objectives, and was only reduced by the onset of skin issues arising from the specific choice of electrode pad used by the CAVA device. All participants who withdrew expressed a desire to continue, and up until withdrawing, individual participant compliance was extremely high. Participants who completed the trial in full provided generally positive feedback regarding their experiences of wearing the device.

During the trial, a minor safety issue was identified relating to the long-term wear and repeated replacement of the device’s electrode pads. As stated above, this issue resulted in several participants withdrawing prematurely from the trial. Following discussions with the CAVA device’s manufacturer, medical experts and electrode pad manufacturers, we have concluded that the issues were mostly caused by *skin stripping*, as would be expected after the repeated removal of a sticking plaster. Prior to the start of the trial, we held a patient engagement event, where we gathered feedback from patients on their experiences of suffering from extended periods of dizziness. The feedback gained from that event suggested that such patients may have a particularly high tolerance to discomfort, due to the severity of the symptoms they frequently endure. Therefore, it is reassuring that *real* patients may consider this to be an issue of secondary importance. No adverse events were reported related to the safety of the device itself, providing confidence that the CAVA device is otherwise safe to wear.

### Limitations of the study

The computer algorithm applied to the trial data has demonstrated that the fundamental frequency of nystagmus (i.e. the number of nystagmus beats per second) can be accurately measured from the CAVA data. In isolation, this is of limited clinical use, as the Slow Phase Velocity (SPV) is the preferred method used by clinicians to quantify nystagmus. The SPV provides a measure of the speed of eye movement in *degrees per second*. In order to do this calculation, ocular excursions have to be measured in degrees, which currently CAVA does not do. The corneo-retinal potential (CRP) recorded by the CAVA device requires calibration to relate it to an absolute position in *degrees*. The CRP has shown to be influenced by many factors, including light, fatigue and diurnal rhythm^31^, and therefore, robust methods of normalising for these differences would have to be developed to allow this association to be made.

While this study does represent a significant advance in the development of the CAVA device, the device itself is currently an advanced prototype. A significant amount of work will be required to develop it from a research tool to a system which could be routinely deployed among real patients in primary or secondary care. Here, we have demonstrated that the system can identify horizontal nystagmus which has been induced visually, but identifying the aetiology of dizziness will also require the identification of up- or downbeat nystagmus, and nystagmus resulting from actual vestibular conditions and other causes. The eye-in-orbit position may also be less consistent when observing real dizzy attacks, as patients are known to vary their gaze in order to reduce the severity of their dizziness^32^, and the effects of this on diagnostic accuracy are currently unknown. We have no reason to suspect that vertical eye movements are not captured at a similar level of detail to horizontal movements, and shortly we will address these points by way of a further trial in dizzy patients (See *Future Work*).

### Implications for the future

The results described here have several implications for both the medical and scientific communities. Firstly, the ability to detect nystagmus provides a promising indication that the cause of a patient’s dizziness may be determined from data captured by the CAVA device. At the highest level, the absence of nystagmus would suggest a cause not of vestibular origin. The long-term data captured by the CAVA device has never been observed before. When applied to patients suffering from dizziness, it is possible that the characteristics of the nystagmus captured may provide sufficient information to assist with the diagnosis of distinct conditions, and may also allow sub-typing, staging, the determination of disease severity, and the monitoring of treatment responses. The detection algorithm used here has already shown to be able to distinguish between left and right-beating nystagmus, a feature of nystagmus which, in patients suffering from dizziness caused by inner ear malfunctions, can suggest the affected ear. A device such as CAVA, if routinely deployed in clinical care, would lead to swifter and more accurate diagnoses, faster access to more accurate treatments, and would reduce unnecessary investigations and consultations.

The data captured during this trial is believed to be the largest dataset of eye movement data ever captured. As well as facilitating further development of our computer algorithms, this data may also be of interest within the areas of sleep analysis and activity detection. The CAVA device also has the potential to provide insight into many medical conditions other than those related to dizziness, such as stroke and sleep medicine, and as such, the device could be used to capture new data in these areas.

### Future work

To further investigate the mechanism of skin trauma and to identify a solution to this safety issue identified during the trial, we have recently started a new trial in which we intend to explore the safety of alternative electrode pads and possible alternatives to the current pad replacement regime. We have identified a number of alternative electrode pads, including some with hypoallergenic electrodes that contain a ‘breathable’ fabric, and using either a wet or a solid hydrogel. Furthermore, we intend to explore whether increasing the duration of wear (i.e. replacing the electrodes less frequently) delays the onset of skin reddening.

Having demonstrated the capability of the device on healthy volunteers, we are also preparing to undertake a further trial on patients suffering from dizziness and vertigo. By deploying the device on such patients, we will collect a wealth of dizziness data which will evaluate the CAVA device’s capability to capture a wider range of nystagmus signals, as well as continuing to evaluate nystagmus detection accuracy, reliability, acceptability and safety. The data we intend to capture may also provide insight into the varying patterns of nystagmus presented by the different dizziness conditions, which have not been systematically observed hitherto.

## Conclusions

The CAVA device was worn by seventeen participants for up to thirty days, who in total captured around 9000 hours of eye and head movement data, totalling 405 days’ worth of data. In this trial, we have evaluated a fully functional medical prototype in a rigorous and challenging manner; our device performs continuous ambulatory monitoring in a non-clinical setting, with minimal intervention from medical or scientific professionals. The data captured by the device was successfully used to identify short periods of visually induced nystagmus with a high degree of diagnostic accuracy. The device was reliable, functioned well as an ambulatory monitor and was tolerated well by the trial participants. Aside from a minor issue related to the repeated removal of the device’s electrode pads, the device was found to be entirely safe. The success of this trial has proven the potential of this system to Fulfil a demonstrated clinical need, and for establishing a new field of medicine; *vestibular telemetry*. These results have provided a good foundation from which to conduct a further study intended to evaluate the system’s diagnostic accuracy among *real* dizzy patients.

## Supplementary information

Supplementary Information

## Data Availability

The dataset generated by this study is available from the corresponding author on reasonable request.
