# Supplementary material for: An investigation into the diagnostic accuracy, reliability, acceptability and safety of a novel device for Continuous Ambulatory Vestibular Assessment (CAVA)
Source: Sci Rep. 2019 Jul 18;9:10452. doi: 10.1038/s41598-019-46970-7 (PMC6639326; doi:10.1038/s41598-019-46970-7)
Supplement: Supplementary file 1 — Supplementary Information [file 41598_2019_46970_MOESM1_ESM.docx]

**Supplementary Information**

**An investigation into the diagnostic accuracy, reliability, acceptability and safety of a novel device for Continuous Ambulatory Vestibular Assessment (CAVA)**

**John S Phillips, Jacob L Newman, Stephen J Cox**

**Supplementary Figure S1.** The trial diary provided to participants of CAVA Clinical Investigation.

**Supplementary Figure S2.** The questionnaire completed by participants of the CAVA Clinical Investigation. Only participants who completed the full trial were asked to complete this questionnaire.
